# Women, poverty and adverse maternal outcomes in Nairobi, Kenya

**DOI:** 10.1186/1472-6874-10-33

**Published:** 2010-12-01

**Authors:** Chimaraoke O Izugbara, David P Ngilangwa

**Affiliations:** 1 African Population and Health Research Center, Box 10787-00100 GPO, Nairobi, Kenya; 2 School of Public Health, University of Witwatersrand, Johannesburg, South Africa; 3 Department of Sociology & Anthropology, University of Uyo, Uyo, Nigeria

## Abstract

**Background:**

The link between poverty and adverse maternal outcomes has been studied largely by means of quantitative data. We explore poor urban Kenyan women's views and lived experiences of the relationship between economic disadvantage and unpleasant maternal outcomes.

**Method:**

Secondary analysis of focus group discussions and in-depth individual interviews data with women in two slums in Nairobi, Kenya.

**Results:**

Urban poor women in Nairobi associate poverty with adverse maternal outcomes. However, their accounts and lived experiences of the impact of poverty on maternal outcomes underscore dynamics other than those typically stressed in the extant literature. To them, poverty primarily generates adverse maternal outcomes by exposing women to exceedingly hard and heavy workloads during pregnancy and the period surrounding it; to intimate partner violence; as well as to inhospitable and unpleasant treatment by service providers.

**Conclusions:**

Poverty has wider and more intricate implications for maternal outcomes than are acknowledged in extant research. To deliver their expected impact, current efforts to promote better maternal outcomes must be guided by a more thorough perspective of the link between women's livelihoods and their health and wellbeing.

## Background

The rich-poor gap in maternal outcomes has been examined largely by means of quantitative data and explained principally in terms of poorer women's reduced chances of receiving prenatal care [1]. During prenatal care, women undergo screening and receive treatment for conditions that could be life-threatening. Poverty hampers women's ability to use otherwise available maternal care services. For instance, lack of resources to pay for transportation could frustrate access to quality care at critical moments. Other accounts have emphasized poorer women's elevated odds for depression; to use alcohol, tobacco, and other harmful substances [2,3]; higher risk for food insufficiency and insecurity and poor feeding practices and habits, resulting in malnutrition and obesity; less access to vitamins and minerals; maternal thinness; decreased blood flow; infections; gestational diabetes; pre-eclampsia; large size for gestational age; fetal macrosomia; and cesarean delivery [4-6]; increased risk for STIs [7]; including HIV: and higher probability to experience closely-spaced births which leads to substantial loss of vital stores of micronutrients; decreased opportunity to restock nutrients; and maternal depletion; morbidity and mortality[8].

But how do poor women themselves view economic disadvantage *vis-a-vis *undesirable maternal outcomes? How, in poor women's views, does poverty engender adverse health outcomes? And what are poor women's lived experiences of the poverty-adverse maternal outcomes link? Prior research has scarcely paid attention to lay views of the relationship between poverty and maternal health outcomes. While studies exist on poor women's views of the factors that constrain their access to formal obstetric care services, very little of these have addressed women's views of the specific ways through which poverty, as a particular and oft-mentioned social determinant of health, impinges on maternal health as a whole. One recent paper, for instance, addressed barriers related specifically to formal emergency obstetric care utilization among poor urban Kenyans women [9]. While it spotlighted poverty as a key inhibitor of urban Kenyan women's use of emergency obstetric care services, the study did not explore lay views of the relationship between economic disadvantage and maternal health as a whole. Yet, it is important to understand 'ordinary' people's viewpoints of the interaction between economic disadvantage and health status. Lay views can expose complexities in the relationships between wellbeing and livelihoods, and thicken contextual understanding of the social and cultural determinants of health. Lay views can also offer researchers the resources with which to more fully contemplate the lives and experiences of individuals affected by real-world problems, understand how human needs nurture new possibilities and alternative practices, and reveal the intricate dynamics and channels through which poverty impacts local people and communities [10].

The current study ponders the direct views of urban poor Kenyan women on the relationship between economic disadvantage and poor maternal outcomes. Focus on urban poor Kenyan women is, on the whole, important. Research shows that they are among the worst hit by adverse maternal outcomes in Kenya. For instance, while the Kenya Demographic and Health Survey of 2003 shows that about 414 maternal deaths occur per 100 000 live births, some poor urban areas of Kenya have maternal mortality figures of 1300 deaths per 100 000 live births[11,12]. Results of recent research in the same slums where the current study was implemented showed a maternal mortality ratio of 706 deaths per 100,000 [13]. There is also ample evidence of the disproportionate representation of urban poor women among the thousands of reproductive-age Kenyan women who suffer short and long-term maternal-related complications and morbidities [12,14,15]^. ^In addressing women's views of the relationship between economic disadvantage and adverse maternal outcomes, this paper thus seeks to enlarge understanding of the role of poverty in maternal outcomes well beyond questions of poor nutrition and under-utilization of appropriate maternal services.

## Method

The current study is a secondary analysis of qualitative data collected in two slums in Kenya. Conducted as part of a larger three-country investigation of maternal health in cities in Ghana, India, and Kenya, the parent study sought to illuminate issues surrounding maternal and child health as well as emergency obstetric care utilization in resource-poor urban contexts. The Kenya study was conducted in two slum settlements in Nairobi: Viwandani and Korogocho. In these two sites, the African Population and Health Research Center (APHRC) operates the Nairobi Urban Health and Demographic Surveillance System (NUHDSS), with about 60,000 registered persons. Among many other issues that emerged from the study, the current paper addresses women's perspectives of the impact of poverty on maternal health. Other themes that have been explored using the rich qualitative data generated in this study include the persistence of homebirths, notions of hospital and home-based deliveries, and lay constructions of barriers to the utilization of emergency maternal care [9,12,16].

The current study relies on data from focus group discussions (FGDs) and in-depth individual interviews (IDIs), held with a variety of poor women. In total, 12 FGDs and 12 IDIs were held. Table 1 below describes the sample and recruitment strategy.

**Table 1:** Study sample

1. Women who had a pregnancy with complications in the two years (2004-2005) preceding the study: In each community, one FGD was held for each of the age groups 12-19; 20-29; and 30-54. Altogether, 6 FGDs were held in the two communities. Participants were recruited through a community survey.
2. Traditional Birth Attendants (TBAs): recruited through key informants (1 FGD was held in each community)
3. Mothers, especially opinion leaders: recruited through the help of key informants. Altogether, 4 FGDs were held in the two communities.
4. IDIs with 12 purposively-selected women who admitted to experiencing at least one episode of pregnancy complication.

Respondents were selected using a multistage sampling design. The first stage was a household survey of reproductive-age women to identify those with experiences of pregnancy-related 'near-misses' and complications in the two years preceding the study. In each study site, three FGD sessions each comprising about eight purposively-selected women were held. These women were identified during the household survey as having suffered pregnancy-related 'near-misses' and complications. Purposive selection ensured the participation of women with varied demographic, reproductive, and maternal health experiences. One FGD session was also held in each slum community with eight purposively-selected women leaders drawn from critical religious, civic, political and cultural publics. Given their popularity in the study communities, we also interviewed TBAs. Two FGDs (one in each community) were held with twenty three TBAs. These TBAs were identified and recruited with the aid of key informants. Further, in-depth individual interviews (IDIs) were held with twelve purposively-selected women reporting personal experiences of pregnancy-related 'near-misses' and complications.

Four different interviewing guides (three FGD guides and one IDI guide) targeting the different categories of respondents defined in Table 1 were used in the study. The guides were developed based on information from the literature as well as survey data from APHRC's maternal health project, and were also pretested during the pilot phase of the study. The interviews were conducted using a focus qualitative interviewing schedule, administered in Swahili (Kenya's national language) by trained female fieldworkers with high-level experience in conducting qualitative interviews. The fieldworkers, who were mostly university graduates or students, were employees of APHRC's NUDHSS at the time of the study. They had an average age of 29 years and were all Kenyans. All discussions were audio-recorded and later transcribed into English. FGDs took place in private settings (a community school classroom, civic hall, or other rented spaces. The IDIs were conducted at the home of the respondents. When this was not ideal or possible, fieldworkers worked with interviewees to agree on an alternative place. In all cases, we insisted on locations and spaces free of the watchful eyes, threat of sanctions, and influence of nonparticipating onlookers and gatekeepers. Interview sessions typically lasted an average of forty-five minutes. Among other things, interviews sought respondents' views regarding women's use of particular types of birthing facilities; their beliefs related to the use of particular birthing services, their knowledge of the factors that affect maternal outcomes; as well as their experiences and perceptions about poverty as it relates to maternal outcomes.

The Ethical Committee of the Kenyan Medical Research Institute approved the research procedures. Informed consent was also obtained from participants before the interviews were conducted. APHRC enjoys widespread rapport in the communities having maintained research presence there for nearly a decade. As a result, every respondent approached to participate in the study assented. Most of the fieldworkers were also residents of the study communities and thus known to the participants.

Using NUDIST software, interview data were simultaneously but separately coded by the authors and a qualitative data coder. Later, the authors and coder met to review the coding outcomes and ensure inter-coder concordance. Data saturation was not an issue because the sample was both small and comprised women with varied reproductive health experiences. A qualitative inductive approach involving thematic examination of the narratives was adopted to analyze the data [17]. This approach is geared toward improving understanding of meanings and messages in narrative data through the continual investigation of the themes emerging from the transcripts for categories, linkages, and properties. Transcripts were also further discussed with participants and colleagues and clarifications made based on their feedbacks and inputs. The categories that emerged were then contrasted with one another to guarantee the distinctiveness and specificity of their properties. In many instances, word-for-word quotations are used to exemplify responses on pertinent issues and themes. To guarantee anonymity, pseudonyms are used in the paper. The findings of the current study have also been presented to participants, community members and scientific audiences; and feedbacks from these presentations have informed the analysis presented here.

## Results

### The respondents

Responding women were from a diversity of socio-demographic and livelihood backgrounds. Their ages ranged from 16 to 70, averaging roughly 39. Livelihoods for the bulk of them were based primarily on informal economic activities: petty trading, manual laboring, and craftsmanship. TBAs, full-time housewives, and women without personal income sources were also in the sample. Among slum dwellers in Nairobi, incomes are generally very low, with slum women often earning more poorly than their men counterparts[18].

Respondents frequently self-reported as married. Only a handful of them were divorced, single, or widowed. While respondents' educational profile indicates that they mostly had primary-level schooling, a substantial number reported secondary-level education. Participants with tertiary-level or without formal education were marginal in the sample. The women we studied self-reported largely as Luos, Kikuyus, Luhyas, and Kambas. The other reported ethnicities were Somali, Taita, Gare, Kisii, Olakaye, Borana, and Kuria. Most respondents self-identified as Christians. They were also nearly uniformly distributed between the two slum sites.

### Perceptions of maternal health in the slums

Study narratives unequivocally underlined the critical importance of maternal health. 'When a mother is unwell, the whole family is unwell'; 'Without a strong and healthy mother, everybody in the household will suffer", responding women regularly admitted. Women's health was described as key to children's survival, household wellbeing, and societal continuity. Good health reportedly equips mothers to more competently care for their children and households, bear healthy children, and contribute positively to family upkeep and wellbeing. Poor maternal health was, in contrast, reported as likely to sap family resources, lead to deficient child care, and foster household poverty. Interlocutors frequently noted that healthy mothers and women contribute more positively to the community, and participated more in neighborhood development and organization efforts.

In general, respondents considered maternal health in the slums to be very poor. 'Many women here have poor health...and many of them die during pregnancy and childbirth'; asserted one woman community leader. The respondents noted that maternal mortality and morbidity was common in their communities. Large numbers of women living in the communities reportedly die or take ill during pregnancy and the post-partum period. Pregnancy loss, fetal deaths, stillbirths, unsafe abortions, and HIV were also said to be very common in the slums. Most interlocutors themselves admitted to having suffered life-threatening maternal health problems; and several of them also knew at least one woman in the community who had a maternal health problem. 'It is common for women here to be sick during pregnancy, sometimes you will see them with swollen legs and others looking really sickly'; observed a responding TBA.

Hemorrhage, anemia, hypertension, malaria, placenta retention, premature labor, prolonged/obstructed labor, and convulsion/seizures (pre-eclampsia) were the commonly-mentioned maternal health problems in the study communities. These problems reportedly often resulted in fetal deaths, premature births, pregnancy loss, and maternal mortality, morbidity, and deformity. In the very apt language of one respondent:

In this community, most people are poor...the women are always sick and sometimes they do nothing about their health because they do not have money to seek treatment. It is common to see women here die from bleeding, convulsion, and premature labor and births. The other day, my neighbor almost died from prolonged labor. The baby died. She was in labor for days... and was delivering at home.

Traditional birth attendants (TBAs); private, missionary, and public formal facilities; itinerant peddlers of western medicines; chemists; herbalists; and religious and magical healers were identified as key providers of maternal health care in the slums. Each provider-type reportedly had its strengths and weaknesses. For instance, the availability of providers and equipment that could make pregnancy and childbearing safer was mentioned as the major benefit of hospital-based care. Hospital-based providers purportedly had the competency to make childbearing safer and hospital-based delivery put women under the care of skilled providers and ensured the ready availability of equipment for managing emergencies and difficult deliveries. Informal providers (e.g., TBAs) reportedly lacked these skills and tools. Martha, a respondent, admitted that hospital-based providers saved her life. She sought delivery services from a TBA and stayed two nights in the TBA's house writhing in labor pains. Finally, the baby arrived feet first. Martha was scared and asked to be transferred to a hospital, but the TBA refused, promising that she could handle the situation. However, Martha recognized she was in grave danger and crawled out of the TBA's house. She was lucky to find a taxi to take her to a hospital in the city. She passed out upon reaching the hospital and remembered waking up with a baby girl by her side. Martha is convinced that she would have died if she had remained at the TBA's home.

Yet, hospital-based deliveries were generally considered to be very expensive and often out of the reach of slum women. Hospital-based maternal care providers were also perceived as harsh and unsympathetic toward poor women. Noted one woman: 'Even those facilities belonging to government or churches and offering free or discounted services, it is not easy for us to make use of them. They may not even ask for anything from you, but ... the whole thing is not easy for us ... You still have to convey yourself there, pay for tests, and buy drugs ... sometimes; we just can't pay for all these because of poverty ... so we go to the TBAs'.

While respondents frequently admitted to the superiority of the hospital as a delivery site, they viewed it primarily as a birthing site for women anticipating or at risk of obstetric emergencies and difficult deliveries. Respondents tended to consider the management of uncomplicated deliveries to be the time-honored role of TBAs, who were depicted as naturally and divinely gifted to assist during deliveries. TBAs' inborn expertise and skills were also viewed as more effectual and reliable than the learned practice of hospital-based providers. One responding woman's view that TBAs were divinely-gifted with the abilities to help women received massive support among the participants. The same approving response greeted the view of a middle-aged FGD participant that "Many TBAs are better than hospital providers when it comes to handling deliveries. It is their work and many of them are really good at it." Another woman also noted: 'They (TBAs) may not be as good as the doctors and nurses, but they help us a lot'.

Self-treatment during pregnancy and the post-partum and self-assisted deliveries were also commonly reported by the women. One woman admitted that she does not go to hospitals or to TBAs for any pregnancy-related conditions. She self-treats by buying medicines from chemists. Another woman reported that she delivered all her last three children assisted only by her teenage daughter.

### Poverty and adverse maternal outcomes

Respondents generally acknowledged their economic disadvantage and vulnerability, commonly commenting that: 'Most of us here are poor'; 'The poorest people in Kenya live here'; 'Only the poor like us live here'; 'Most people you see here are poor'; 'Here in the slums, you will find the poorest of the poor in Kenya'. Responding women widely associated poverty with key social problems, including insecurity, deprived housing conditions, poor nutrition, unsafe abortion, inability to educate one's children, alcoholism, drug use, crime, delinquency etc.

The narratives we collected strongly linked poverty and negative maternal outcomes, casting poverty as the major killer of women in the slums and a key hindrance to women's wellbeing and survival during pregnancy and the post-partum period. Of course, they linked poverty to decreased utilization of appropriate antenatal care and delivery services as well as to poor nutrition. Due to poverty, slum women were reportedly often undernourished, scarcely used quality maternal services, and delivered at home. Poor nutrition also reportedly left women with poor quality blood and insufficient nutrients to go through pregnancy and the period surrounding it. Starving, weak, or underfed mothers were said to be common sight in the slums. Such women usually die, get sick, or suffer complications during pregnancy and the post-partum. Poverty was also said to hamper slum women's access to quality care. Facilities that offer good services in the slums were often privately-run and charged exorbitant prices. They were thus beyond the reach of poor women. The cost of reaching quality public maternal health services located outside the slums also emerged as a major hindrance to women's access to quality care. Several responding women also frequently implicated poverty in their own problematic maternal outcomes and for the maternal complications of other women personally known to them.

Respondents admitted to seeking homebirths because of their affordability. During homebirths, women did not have to pay for transportation, registration, laboratory, and other costs, including bribes reportedly offered to formal providers to facilitate services. They also did not have to pay for supplies such as transfusion blood, syringes, needles, drugs, and sanitary materials, which would be incurred during a hospital stay. Josephina, a mother of four, brought into bold relief the implications of poverty for women's uptake of hospital-based maternal services. She gave birth to her first baby in a public health facility in Nairobi at a time when she was unemployed and her husband did not have a stable job. Josephina recalled going to the hospital numerous times for consultations and says that she spent a lot of money during the period. There were days she would trek to the hospital due to lack of transport fare. In addition to paying various amounts for minor services, she also regularly bribed hospital staff to ensure that she would receive swift attention in the hospital. Josephina also paid in advance for blood that she would be transfused with, although she never received any at delivery and was never refunded her money. She was also requested to buy her own supplies (e.g., sanitary towels, cotton wool, and syringes), which were deposited in the hospital. Labor began for Josephine at night and her husband had to pay about 600 Kenyan shillings ($10 U.S.) to hire a taxi to transport her to the hospital. While acknowledging the risks in homebirths, Josephine says, "unlike homebirths, hospital-based deliveries make poor people poorer...'

However, in the context of the slum, the women maintained that poverty engenders undesirable maternal outcomes not primarily by preventing women's access to quality nutrition and maternal services, but by exposing them to extremely heavy workloads during pregnancy, to intimate partner violence, as well as to inhospitable and poor treatment by service providers. In what seemed like the mind of most our interlocutors, 32-year-old Anna asserted that 'Women here are poor, but they also devotedly attend antenatal services and they try to eat well during pregnancy...but poverty still causes us to have problems during pregnancy because of other things'. In her longer narrative, Anna suggested that among slum women, poverty operates through dynamics, other than restricting women's access to quality services and nutrition, to cause adverse health outcomes among slum women.

The heavy workload which poverty reportedly pushes women into during pregnancy and the post-partum period was a prominent explanation offered for adverse maternal outcomes among slum women. For many responding women themselves, it was the heavy workload which they perform during pregnancy and the postpartum period that caused them adverse maternal experiences. Due to poverty, slum women reportedly continued to do so much hard work during pregnancy and the period surrounding it. They would work in construction sites as head-carriers and loaders, stay out late selling their wares, or go from door to door looking for work, etc. Hard work during pregnancy and the period surrounding it reportedly sapped women's energy and blood, leaving them weak and fragile. For the respondents, women worked harder during the period of pregnancy in order to save enough money to prepare for birthing. To be able to cater for their babies, some women also reportedly resumed heavy work immediately after delivery. Julie blamed heavy workload during her pregnancy for the severe anemia she suffered. Always exhausted, Julie said she never rested adequately and blamed it all on poverty: she needed to save enough money to prepare for the baby and the time she will spend at home after delivery. As she continued to toil during this precarious period, Julie got burnt out, and became anemic. Aloeci, a 27-year-olf mother, who reported that she nearly died 5 days after her delivery also linked her 'near-miss' experience to heavy workload. She worked as a cleaner till two weeks before her delivery and resumed her job 4 days after delivery. It was on her first day at work after delivery that she suffered heavy bleeding. 'I had to start working immediately or I would starve with my children. I would have stayed at home and rested. But now I needed money. I nearly died'. Women's workload also reportedly increased during pregnancy and soon after birthing because husbands never bring home enough for the upkeep of households. Some men also reportedly run away when their wives become pregnant. In the case of Moriga, her husband chased her out of the house when she became pregnant, saying he did not have the resources to have another child. To prepare for the delivery of the baby, Moriga said she took a job as a bar tender. However, she often worked late, rested little, and stood for long periods. One day, out of exhaustion, she collapsed at work and lost the baby.

Another frequently reported means through which poverty promoted adverse maternal outcome in the slums was by exposing women to experiences of intimate partner violence during pregnancy and the period surrounding it. Owing largely to poverty, hardship, and unemployment, men in the slums were reported as often extremely frustrated and desperate, and which lead them into violent behaviors toward their wives. The physical abuse of women by their male partners was reported as common in the slums, and held as a key issue in slum women's adverse maternal outcomes. Wanjiru admitted that she lost her pregnancy to the constant beating she received from her jobless and alcoholic husband. 'He used to be a good man before, but when he lost his job; he became frustrated and beat me all the time. Here, men drink a lot and go home to beat their wives". She asserted. In her longer narrative, Wanjiru noted that most poor men in the slum get worried when their wives get pregnant. 'Because of the burden children bring, they get frustrated and often vent their frustration on their wives. It is not uncommon for men to kill their wives here when they are pregnant. That's what poverty causes here'. One unemployed man reportedly beat his wife until he gave birth prematurely. Another alcoholic and jobless husband allegedly kicked his pregnant wife in the stomach, killing her. A respondent had a neighbor who started beating his pregnant wife when he lost his job, until she suffered a miscarriage. There was also an account of a woman who experienced 4 stillbirths following 5 years of unrelenting physical mistreatment in the hand of an abusive, alcoholic, and jobless husband.

A third major way mentioned by the women through poverty reportedly engendered adverse maternal outcomes among slum women was by exposing them to inhospitable treatment by service providers. Respondents agreed that though while most slum women were often very willing to use modern maternity and delivery services, they usually suffered poor treatment when they presented in these facilities. As the women's narratives stoutly implied, their poverty was to blame for this. Providers reportedly were uncharitable toward poor health seekers, often abandoning them or ignoring them when they present at formal facilities. Among other confirmatory narratives, a 27-year-old Korogocho mother observed that when poor women walk into hospitals with their inexpensive dresses, they are easily identified by nurses and doctors, some of whom even act towards them as if they smelled. She said, "Some of them are so wicked that they will not pay you any attention until you are dying''. Providers were said to be deferential toward well-off care-seekers, who offer tips and bribes. Poor women who cannot afford to give bribes and tips have to wait for long periods of time before receiving attention. Some poor women reportedly only receive attention when they faint in waiting lines or are almost dying. 'Sometimes you go to the clinic and you are in labor; they will just ignore you because you are poor. They know there is nothing you will give them; so they only come to you when you are on the floor, dying in your own pool of blood or water'. Many women here have problems because they are poor and providers mistreat them'. The poor treatment received by poor women when they present at the hospital reportedly also push them to use less efficacious services, such as TBAs. Martha (aged 34) also noted, "Child delivery costs a lot in the hospital and when poor people like us go there, we are treated shoddily''.

## Discussion and Conclusion

This study addressed poor Kenyan women's views and lived experiences of the link between poverty and adverse maternal outcomes. Its major limitations are that it is a secondary analysis, which confined us to an existing dataset; relies on information gathered from women who are not typical of a national or local sample of poor Kenyan peoples, and is characterized by a certain ethnographic skinniness as long-term field observations did not accompany the interviews. The small size of the sample also raises the possibility that data saturation may not have been achieved, which implies that the themes and issues under consideration may not have been exhaustively treated. Yet, it contains a number of important findings. For instance, like the women studied by other scholars, our participants underscored the importance of maternal health [9,19,20]. They recognized healthy mothers and women to be key to the wellbeing of children, households, and communities; described their communities as impoverished and characterized by very high levels of maternal mortality and morbidity; and were knowledgeable about the maternal health problems which face women in their community. Responding women's robust awareness about maternal health problems presents an important resource for current efforts to foster change. Women's views of the health problems affecting them are critical in the drive towards sustainability in maternal healthcare delivery[9]. Such information can assist program implementers create more responsive and acceptable interventions. They can also contribute to the process of identifying important questions and relevant outcomes, and support the implementation of research findings [21].

Study respondents admitted to the many adverse impact of poverty on their lives, including the high-levels of morbidity and mortality in their communities. According to them, however, among slum women, poverty produced adverse maternal outcomes not primarily by hindering adequate nutrition and the utilization of appropriate maternity services among women. Rather, they argued that in the slums, poverty engenders adverse maternal outcomes largely by compelling pregnant and puerperal women to do heavy workloads; exposing them to experiences of intimate violence; and rendering them vulnerable to inhospitable treatment by service providers when they present for care.

The potential of poverty to particularly expose women to heavy workloads during pregnancy and the period surrounding it has yet to gain topicality in the literature on adverse maternal outcomes. However, the women we studied held heavy workloads during pregnancy as the key means through which poverty engenders adverse maternal outcomes among them. They generally noted that they worked harder during pregnancy in order to earn and save enough money to prepare for delivery. Maternal depletion syndrome, a major predictor of maternal and child health, has been linked to heavy workloads [22]. Among poor households, pregnant women often take on additional workloads to prepare for birthing [23]. Mebrahtu reports that in several African communities, poverty forces women to work untiringly during pregnancy and immediately afterwards [24]. The potential of poverty to particularly expose women to heavy workloads during pregnancy and the period surrounding it is a neglected dynamic which current efforts to reduce adverse maternal outcomes in poor communities must begin to recognize.

Slum men were also reported as often physically abusive towards their wives during pregnancy and the post-partum. Intimate partner abuse is very widespread in sub-Saharan Africa and occurs with increased regularity and severity among economically-disadvantaged pregnant women [25]. Costs associated with pregnancy may add to the exasperation and despondency of poor men, predisposing them to violent behavior [26]. Campbell associates intimate partner violence (IPV) with adverse maternal and fetal outcomes. Men's role in ensuring quality maternal outcomes in poor communities must go beyond their ability to recognize danger signs in pregnancy [27]. Men need help to realize how poverty may drive them to behave in ways that endanger the lives of their wives or female partners.

Providers were also reportedly very uncharitable toward slum women because they were poor. When poor women present at facilities, providers would reportedly abandon them, not listen to them, not ask them important questions, and not attend to them. Such mistreatments reportedly contributed to fatalities among slum women and discouraged some of them from seeking formal providers. Poor patient-provider relationships and provider inattention to health seekers' needs are foremost barriers to the uptake of formal care services and frequently-mentioned factors in poor maternal outcomes in developing societies^. ^Caregivers lose a unique opportunity to contribute to the elimination of health disparities by treating low-income women unfairly [28]. Better patient-provider relationship is a practical area of focus towards improving maternal outcomes for poor women and households. In the current study, TBAs were considered accessible and affordable; they treated women more kindly and thus enjoyed more patronage. This is important and suggests the critical role of TBAs in maternal health delivery among poor communities. In this respect, TBAs have a relevance which can be strengthened through extended training as well as back-up support from formal providers [16].

To conclude, our respondents recognized poverty as a major killer of slum women and a key hindrance to women's wellbeing and survival during pregnancy and the period surrounding it. Their lived experiences of the impact of poverty on maternal outcomes suggested however, that poverty engenders adverse maternal outcomes largely by driving pregnant and puerperal mother into heavy workloads; exposing them to experiences of intimate violence; and making them vulnerable to hostile treatment by service providers. Overall, the evidence presented here is critical for social and policy action aiming to improve the maternal outcomes among poor women. It suggests wider and more complex implications of poverty for maternal outcomes than are readily acknowledged in extant research, raising urgent need for efforts aiming to promote better maternal outcomes to be guided by a vigorous understanding of the multiplicity of ways through which women's livelihoods mediate their health outcomes.

## Competing interests

The authors declare that they have no competing interests.

## Authors' contributions

All authors were involved in conceptualizing the paper and analyzing the data. COI wrote the first draft and DPN reviewed it and made suggestions for improving it. Both of us read and approved the final manuscript.

## Pre-publication history

The pre-publication history for this paper can be accessed here:

http://www.biomedcentral.com/1472-6874/10/33/prepub
